# 2622. Recent or concurrent rhino/enterovirus infection with respiratory syncytial virus associated with decreased symptoms and medical care use in healthy children, PREVAIL, 2017–2020

**DOI:** 10.1093/ofid/ofad500.2235

**Published:** 2023-11-27

**Authors:** Maria Deza Leon, Adam E Gailani, Shannon C Conrey, Dorcas Washington, Allison Burrell, Zheyi Teoh, Meredith L McMorrow, Daniel C Payne, Ardythe L Morrow, Mary A Staat

**Affiliations:** Cincinnati Childrens Hospital, Cincinnati, Ohio; Cincinnati Children's Hospital Medical Center, Cincinnati, Ohio; University of Cincinnati College of Medicine, Cincinnati, Ohio; University of Cincinnati, Cincinnati, Ohio; Cincinnati Children's Hospital and Medical Center, Cincinnati, Ohio; Cincinnati Children's Hospital Medical Center, Cincinnati, Ohio; CDC/NCIRD/CORVD/SPB, Atlanta, GA; US Centers for Disease Control and Prevention, Atlanta, GA; University of Cincinnati College of Medicine, Cincinnati, Ohio; Cincinnati Children’s Hospital Medical Center, Cincinnati, Ohio

## Abstract

**Background:**

Respiratory syncytial virus (RSV) infections are common in children, resulting in thousands of hospitalizations each year. Viral coinfections are frequently detected during RSV infection, but the effect of these coinfections is poorly understood, and most studies only include hospitalized children. We examined the effect of the most frequently identified co-infection, rhinovirus/enterovirus (RE), on symptoms status and medical care use in a birth cohort of healthy children with RSV.

**Methods:**

PREVAIL is a CDC-funded, 2-year birth cohort (2017–2020) in Cincinnati, OH of healthy, full-term infants. Weekly, mid-turbinate nasal swabs were tested using the Luminex Respiratory Pathogen Panel. RSV infection was defined as a swab positive for RSV (A or B). C Concurrent coinfection was defined as detection of RE at the time of RSV infection, prior RE infection was defined as a swab positive for RE up to 14 days prior to RSV infection. Symptomatic infection was defined as presence of cough or fever reported by the mother *via* text survey or abstracted from the child’s medical record and medical care use was defined as receiving medical care or not. Odds of symptomatic infection or medical care use with RSV were calculated using a generalized estimating equation adjusted for the child’s age at infection, type of insurance, coinfection with RE and sex.

**Results:**

Overall, 128 RSV infections were identified in 101/245 (41%) children; 89 (69%) were symptomatic, 59 (46%) were medically attended, 4 (3%) were hospitalized. Concurrent co-infection with RE occurred in 20% (*n*=26) and 31% (*n*=40) had a RE coinfection between 0-14 days prior to the RSV episode. Coinfection with RE, whether concurrent (aOR 0.29, 95%CI 0.10-0.82) or prior (aOR 0.18, 95%CI 0.07-0.45) was associated with decreased odds of symptomatic disease. Concurrent (aOR 0.19 95%CI 0.06-0.60) or prior (aOR 0.25, 95%CI 0.11, 0.55) RE coinfection was also associated with decreased odds of medical care use.

**Conclusion:**

RSV infections were common in the PREVAIL cohort, most infections were symptomatic with nearly half medically attended. Coinfection with RE 0-14 days prior to RSV infection was associated with decreased odds of both symptoms and medical care use.

**Disclosures:**

**Mary A. Staat, MD, MPH**, CDC: Grant/Research Support|Cepheid: Grant/Research Support|Merck: Grant/Research Support|NIH: Grant/Research Support|Pfizer: Grant/Research Support|Up-To-Date: Honoraria

